# The influence of post-processing software on quantitative results in 4D flow cardiovascular magnetic resonance examinations

**DOI:** 10.3389/fcvm.2024.1465554

**Published:** 2024-09-27

**Authors:** Ralf F. Trauzeddel, Maximilian Müller, Aylin Demir, Stephanie Wiesemann, Elias Daud, Sebastian Schmitter, Darian Viezzer, Thomas Hadler, Jeanette Schulz-Menger

**Affiliations:** ^1^ ECRC Experimental and Clinical Research Center, Charité - Universitätsmedizin Berlin, Corporate Member of Freie Universität Berlin and Humboldt-Universität zu Berlin, Berlin, Germany; ^2^ Working Group on Cardiovascular Magnetic Resonance, Experimental and Clinical Research Center, A Joint Cooperation Between the Charité—Universitätsmedizin Berlin and the Max-Delbrück-Center for Molecular Medicine, Berlin, Germany; ^3^ DZHK (German Center for Cardiovascular Research), Berlin, Germany; ^4^ Department of Anesthesiology and Intensive Care Medicine, Charité Campus Benjamin Franklin, Charité - Universitätsmedizin Berlin, Corporate Member of Freie Universität Berlin and Humboldt-Universität zu Berlin, Berlin, Germany; ^5^ The Cardiology Department, Galilee Medical Center, Azrieli Faculty of Medicine Bar-Ilan University, Nahariya, Israel; ^6^ Physikalisch-Technische Bundesanstalt (PTB), Braunschweig, Berlin, Germany; ^7^ Department of Cardiology and Nephrology, HELIOS Klinikum Berlin Buch, Berlin, Germany

**Keywords:** cardiovascular magnetic resonance imaging, 4D flow CMR, phase-contrast CMR, post-processing, quality assurance, reliability

## Abstract

**Background:**

Several commercially available software packages exist for the analysis of three-dimensional cine phase-contrast cardiovascular magnetic resonance (CMR) with three-directional velocity encoding (four-dimensional (4D) flow CMR). Only sparse data are available on the impact of these different software solutions on quantitative results. We compared two different commercially available and widely used software packages and their impact on the forward flow volume (FFV), peak velocity (PV), and maximum wall shear stress (WSS) per plane.

**Materials and methods:**

4D flow CMR datasets acquired by 3 Tesla magnetic resonance imaging of 10 healthy volunteers, 13 aortic stenosis patients, and 7 aortic valve replacement patients were retrospectively analyzed for FFV, PV, and WSS using two software packages in six analysis planes along the thoracic aorta. Absolute (AD) and relative differences (RD), intraclass correlation coefficients (ICC), Bland–Altman analysis, and Spearman's correlation analysis were calculated.

**Results:**

For the FFV and PV in healthy volunteers, there was good to excellent agreement between both software packages [FFV: ICC = 0.93–0.97, AD: 0.1 ± 5.4 ml (−2.3 ± 2.4 ml), RD: −0.3 ± 8% (−5.7 ± 6.0%); PV: ICC = 0.81–0.99, AD: −0.02 ± 0.02 ml (−0.1 ± 0.1 ml), RD: −1.6 ± 2.1% (−9.3 ± 6.1%)]. In patients, the FFV showed good to excellent agreement [ICC: 0.75–0.91, AD: −1.8 ± 6.5 ml (−8.3 ± 9.9 ml), RD: −2.2 ± 9.2% (−13.8 ± 17.4%)]. In the ascending aorta, PV showed only poor to moderate agreement in patients (plane 2 ICC: 0.33, plane 3 ICC: 0.72), whereas the rest of the thoracic aorta revealed good to excellent agreement [ICC: 0.95–0.98, AD: −0.03 ± 0.07 (−0.1 ± 0.1 m/s), RD: −3.5 ± 7.9% (−7.8 ± 9.9%)]. WSS analysis showed no to poor agreement between both software packages. Global correlation analyses revealed good to very good correlation between FFV and PV and only poor correlation for WSS.

**Conclusions:**

There was good to very good agreement for the FFV and PV except for the ascending aorta in patients when comparing PV and no agreement for WSS. Standardization is therefore necessary.

## 1 Introduction

Three-dimensional (3D) cine (time-resolved) phase-contrast cardiovascular magnetic resonance (CMR) with three-directional velocity encoding [four-dimensional (4D) Flow CMR] enables the analysis of qualitative and quantitative blood flow parameters in the heart and blood vessels in a multidirectional way and may therefore improve the understanding of hemodynamics in healthy volunteers and several cardiovascular pathologies (1, 2). As it enters the clinical arena, quality assurance including standardized acquisition and analysis techniques becomes an essential factor in the dissemination of this promising technique as it constitutes the basis for clinical studies and the evaluation of patients. This is especially important as it has been shown that multiple confounders may influence the quantitative results of 4D flow CMR datasets, e.g., different field strengths, sequences, and MR vendors (3, 4). Deviations in the quantified outcome due to different implementation details across post-processing software are another potential source of error-limiting comparability. This would prohibit reliable clinical decision-making and may lead, in the extreme, to the wrong therapy and ultimately harm patients. For measurements with two-dimensional flow CMR, which is currently still the gold standard technique in CMR for the quantification of shunts and valvular regurgitation, it has been shown that different commercially available software solutions have no impact on stroke volume quantification but have a potential influence on the measurement results of peak velocities (PVs) (5). Recently, Oechtering et al. compared basic and advanced 4D flow CMR hemodynamic parameters using four different software packages in healthy volunteers (6). Only two software solutions yielded equivalent results regarding stroke volume, peak flow, and vessel area. However, for the two other software packages and quantitative parameters, e.g., peak velocities and wall shear stresses (WSSs), no equivalency was present. Burkhardt et al. also compared net flow volumes in the ascending aorta and main, right, and left pulmonary arteries in 47 biventricular congenital heart disease patients using four different software packages (7). They could show good agreements with little bias for all the software programs analyzed and concluded that they could all be used for flow assessment in patients. However, a comparative analysis for peak velocities and wall shear stresses in patients with cardiovascular pathologies is pending.

Therefore, the aim of this study is to compare the results of two different commercially available software packages and their impact on different hemodynamic parameters, including forward flow volumes (FFVs), PVs, and maximum WSSs in healthy volunteers and patients with aortic stenosis (AS) and after aortic valve replacement (AVR).

## 2 Materials and methods

### 2.1 Study design

This study was designed as a retrospective analysis of 4D flow CMR datasets (3, 8). All procedures involving humans were conducted in accordance with the ethical standards of the institutional research committee and the 1964 Declaration of Helsinki and its later amendments. The original studies were approved by the local ethics committee at Charité—Universitätsmedizin Berlin (EA1/258/12, date of approval 30 May 2014; and EA1/135/17, date of approval 27 July 2011) and registered at ISRCTN (ISRCTN37755721, registration date 15 March 2018; and ISRCTN17935517, registration date 07 August 2018). Informed written consent was prospectively obtained from all study participants at that time. Ethical approval, registry, and informed consent for the current analysis of the data were waived due to the retrospective nature of the study.

### 2.2 Study population and image acquisition

Datasets of 30 subjects including 10 healthy volunteers (3), 13 AS patients, and 7 with AVR from published studies were retrospectively analyzed (8). Inclusion and exclusion criteria are published in the study literature (3, 8). In short, in the healthy volunteers study, only individuals with no known cardiovascular risk factors or any history of cardiac diseases as well as normal right and left ventricular and valvular function based on CMR findings were included (3). In the patients study, participants with moderate or severe AS were initially prospectively recruited and then followed up after 4.4 ± 1.5 years (8). All participants underwent a 3 T CMR examination (Magnetom Verio, Siemens Healthineers, Erlangen, Germany) using a 32-channel receiver coil and the following scan parameters: echo time = 2.6 ms, repetition time = 5.1 ms, temporal resolution of 40.8 ms, bandwith = 450 Hz/pixel, imaging acceleration using k-t GRAPPA with a reduction factor of *R* = 5, net acceleration factor of 4.17, reference lines = 20, nominal flip angle *α* = 7°–9°, field of view = 360 × 270 mm^2^, phase encoding direction = anterior–posterior, number of slices = 32, and encoding velocity = 1.5–2.5 m/s. A respiratory navigator placed over the lung–liver interface, in combination with prospective electrocardiogram (ECG) gating, was used. The entire thoracic aorta was acquired using a sagittal oblique volume.

### 2.3 Image analysis

Post-processing was conducted according to local standard operating procedures (SOP) using CAAS MR Solutions version 5.2.1 (Pie Medical Imaging BV, Maastricht, The Netherlands) (software 1) and Circle CVI 42 version 5.13.7 (Circle Cardiovascular Imaging Inc., Calgary, Alberta, Canada) (software 2), as published previously (4). In all datasets, correction for Maxwell fields was automatically applied online during image reconstruction after acquisition by the MR system (9). Three quantitative hemodynamic parameters were quantified and compared: FFV, PV, and WSS per plane.

#### 2.3.1 Software 1

**Figure 1:**
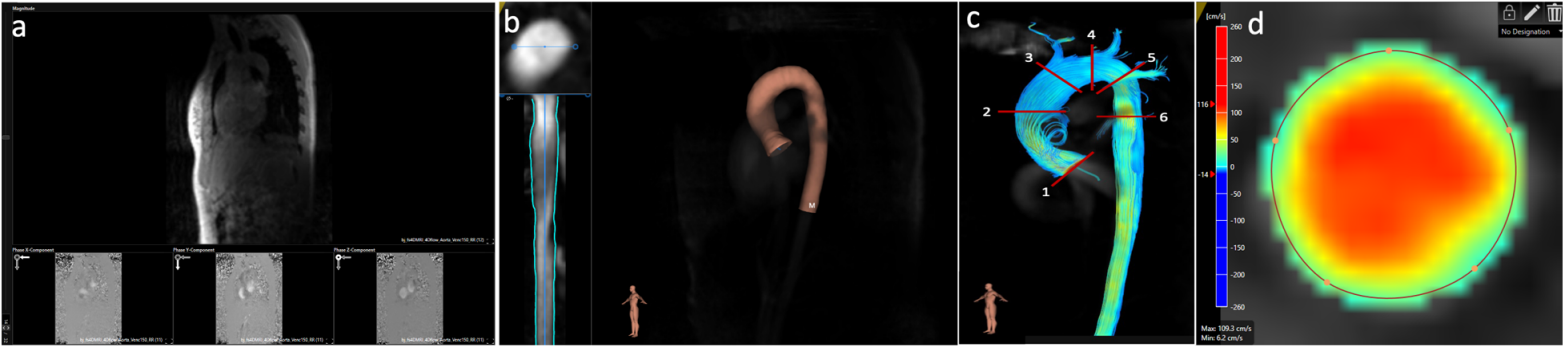
Post-processing of 4D flow CMR data using software 1. **(a)** Aliasing and background phase offset correction. **(b)** Placement of the vessel centerline along the thoracic aorta. **(c)** Positioning of the six analysis planes. **(d)** Manual correction of the lumen contour in every cardiac phase using a velocity mask overlay beside the magnitude images.

Post-processing was performed based on the exported DICOM images as described previously (4). In short, after background phase offset and aliasing correction, a centerline was placed along the thoracic aorta with six analysis planes placed perpendicularly to the aorta at the (1) sinotubular junction, (2) at the mid-ascending aorta, (3) proximal to the origin of the brachiocephalic trunk, (4) proximal to the origin of the left subclavian artery, (5) at the beginning of the descending aorta, and (6) in the descending aorta at the same height level as the second plane (Figures 1a–c) (10, 11). After placement of the planes, automatically generated contours of each one were manually corrected for each phase of the heart cycle to align them to the aortic vessel wall. If residual aliasing was present despite initial correction in the region of an analysis plane, this plane was excluded from further analysis. The FFV was defined as the volume flowing through one plane in the forward direction over the entire heart cycle, and PV was defined as the highest velocity occurring in one pixel within the aorta in one single cardiac phase. As WSS values are provided for 90 circumferential segments in each phase, the mean WSS value was calculated over all segments for each phase. Out of these calculated values, the maximum WSS is given by the highest value.

#### 2.3.2 Software 2

**Figure 2:**
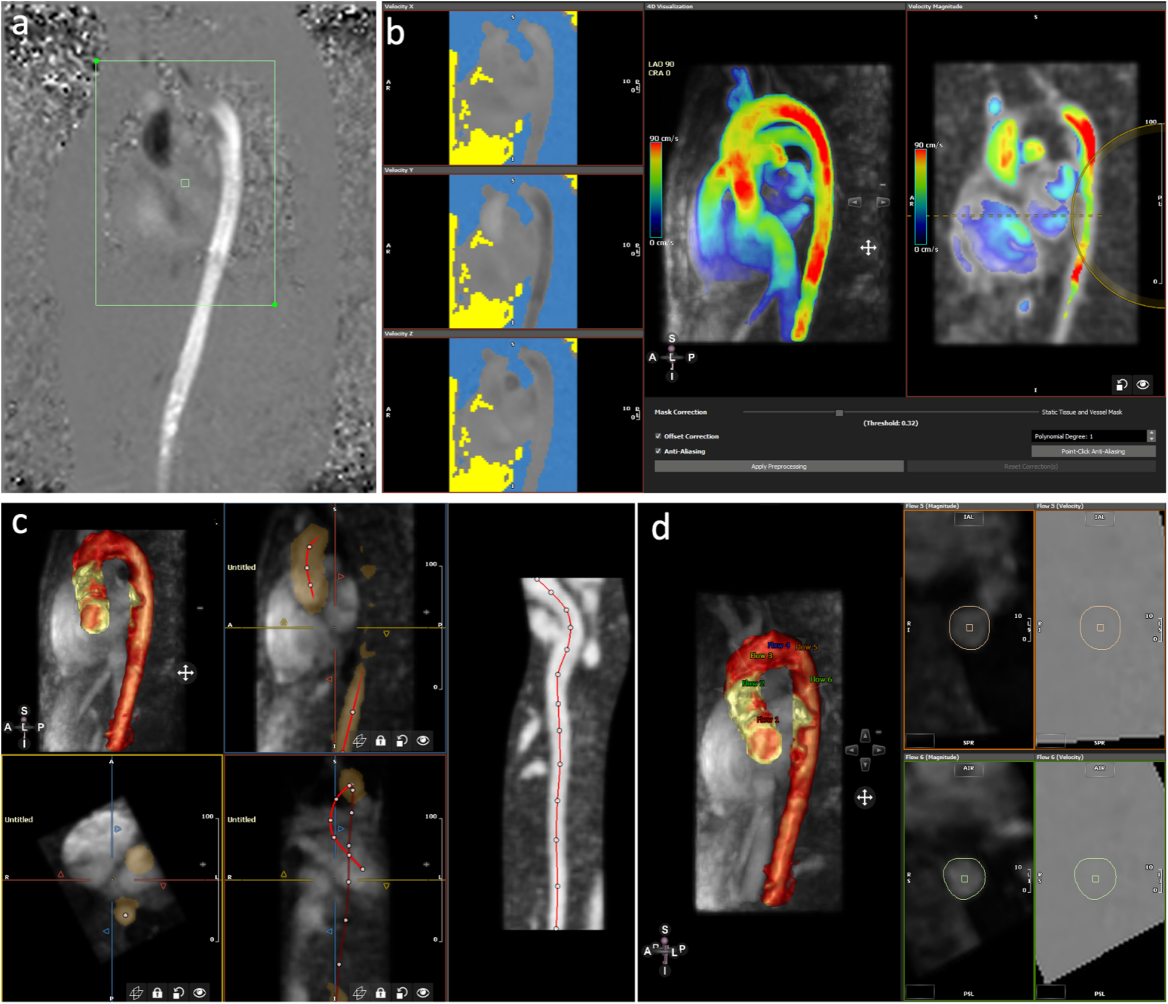
Post-processing of 4D flow CMR using software 2. **(a)** Selection of the area of interest of the acquired image. **(b)** Aliasing and background phase offset correction. **(c)** Placement of the vessel centerline along the thoracic aorta. **(d)** Positioning of the six analyses planes and manual correction of the lumen contour in every cardiac phase.

The entire thoracic aorta, the proximal part of the three supra-aortic vessels and their origin from the aortic arch, and the beginning of the abdominal aorta (Figure 2a) were segmented as the region of interest. If residual aliasing was present in the region of interest within an analysis plane despite initial correction, this plane was excluded from analysis, as in software 1. In addition, background phase offset correction was applied (10, 11) (Figure 2b). For segmentation purposes, a vessel centerline was placed in the thoracic aorta starting at the aortic valve and ending below the diaphragm (Figure 2c). Along the centerline, six planes were placed along the thoracic aorta as in software 1. After placement of the planes, contours of each one were manually corrected as described for software 1 (Figure 2d). The FFV and PV were defined as in software 1. For maximum WSS, one value was provided for each phase per plane. Thus, maximum WSS was defined as the maximum value out of all values exported in one plane.

### 2.4 Statistical analysis

Quantitative data are displayed as mean ± standard deviation (SD) and categorical data as absolute and relative frequencies. For every quantitative parameter, absolute and relative differences with their respective SD between both post-processing software packages were calculated as described previously (6). For relative differences, results from both software packages were divided by the means of the measurement results from both software solutions. Intraclass correlation coefficient (ICC) analyses with a two-way mixed model and absolute agreement were performed for equivalence testing. ICC values were interpreted in the following way: >0.9, excellent; 0.75–0.9, good; 0.5–0.75, moderate; and <0.5, poor (12). Bland–Altman plots were used to assess the bias (mean difference) and 95% limits of agreement of different measurements per plane. Correlation analyses were carried out using Spearman's correlation analysis over all planes. Statistical analysis was performed using GraphPad Prism version 9 for Windows (GraphPad Software, San Diego, CA, USA) and SPSS Version 29 (IBM, Armonk, NY, USA).

## 3 Results

### 3.1 Study cohort and image quality

Demographics of the study participants are displayed in Table 1. In the healthy volunteer cohort, all 60 planes in total could be analyzed. In the patient cohort, 42 out of 120 planes were not included in the final analysis due to residual aliasing despite correction, as mentioned in the Materials and methods section, with one patient displaying aliasing in all analysis planes throughout the aorta. This was especially marked in plane 1, where in all but one patient aliasing was present. Therefore, no results are given for plane 1 in the patient cohort. Moreover, in one patient in software 2, no FFVs were displayed in the last three analysis planes.

**Table 1:** Baseline characteristics of the study participants.

	Healthy volunteers	Patients
*n*	10	20
Sex (female/male)	6/4	6/14
Age (years)	33 ± 9	63 ± 15
Height (cm)	170.9 ± 10.1	172.0 ± 8.8
Weight (kg)	65.2 ± 10.0	79.6 ± 13.0
Body mass index (kg/m^2^)	22.2 ± 1.7	26.8 ± 3.1

Values are presented as mean ± standard deviation.

### 3.2 Forward flow volumes and peak velocities

**Figure 3:**
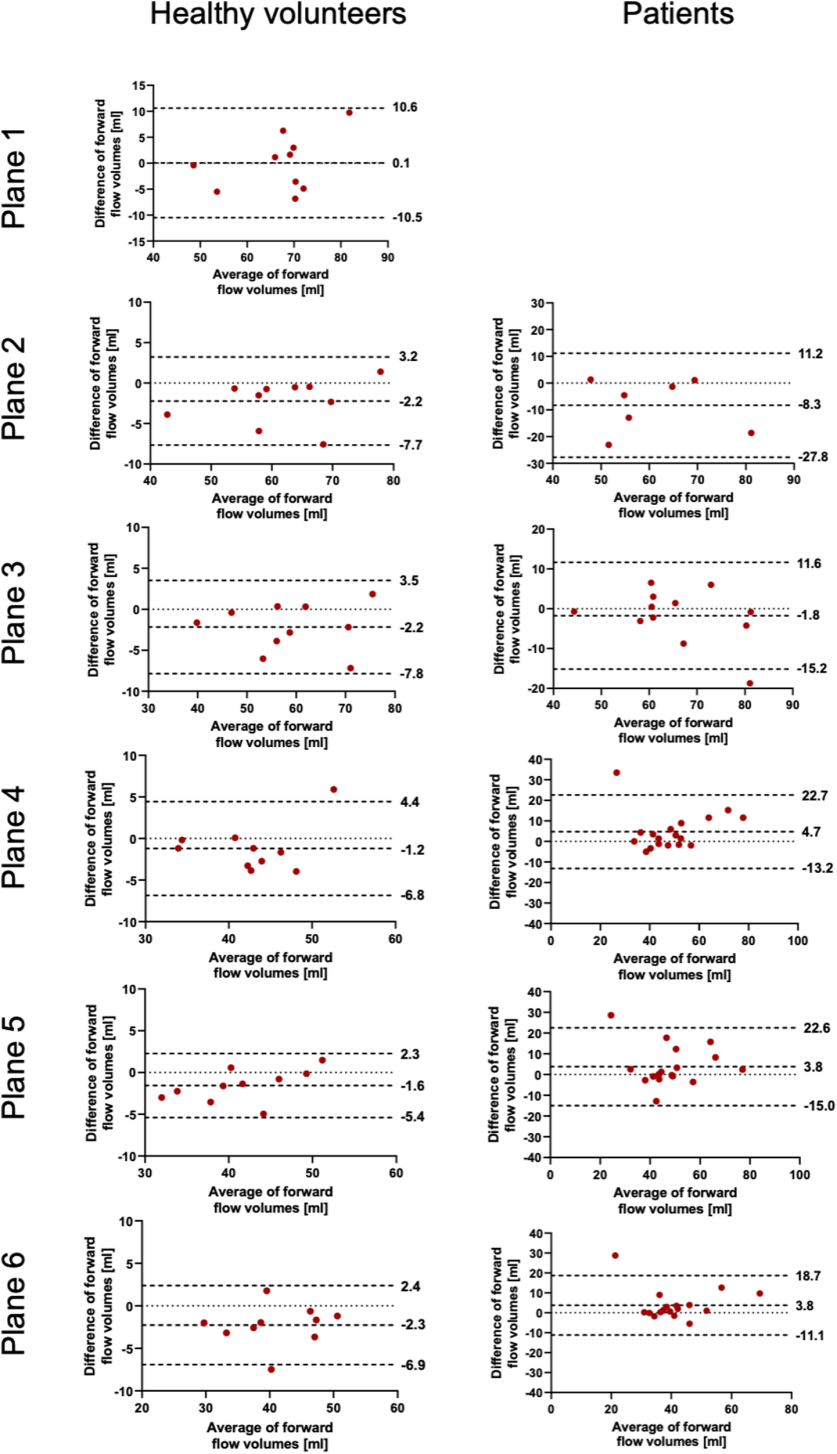
Bland–Altman plots for the plane-wise comparison of forward flow volumes for healthy volunteers and patients. The upper and lower dotted bold lines indicate the 95% limits of agreement. The middle dotted bold line indicates the mean difference.

**Figure 4:**
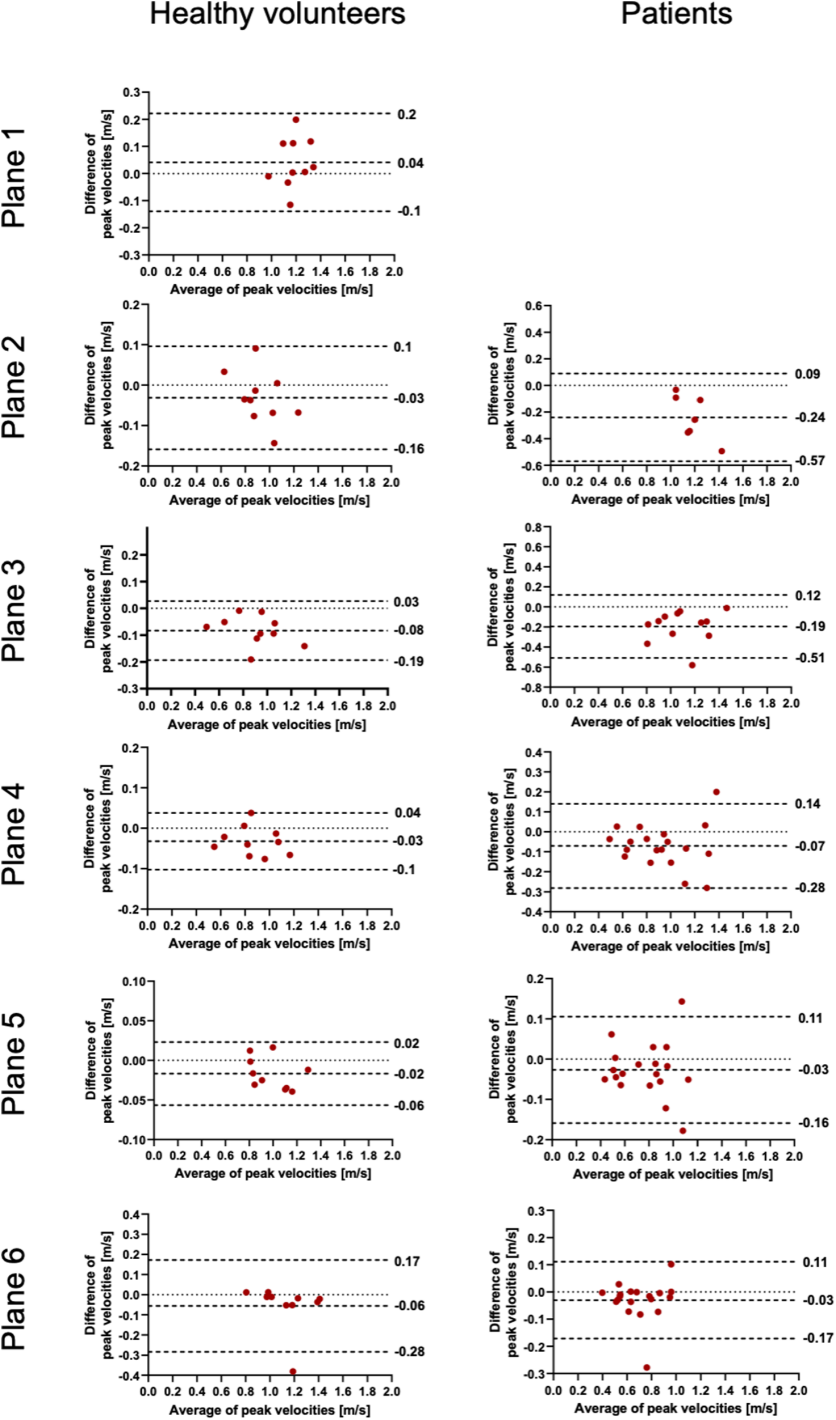
Bland–Altman plots for the plane-wise comparison of peak velocities for healthy volunteers and patients. The upper and lower dotted bold lines indicate the 95% limits of agreement. The middle dotted bold line indicates the mean difference.

The results for the FFV and PV are displayed in Tables 2, 3. The Bland–Altman and correlation analyses for all planes are combined in Figures 3, 4, 6. Correlation analyses for the individual planes are displayed in Supplementary Figures 1 and 2 for the FFV and PV, respectively. In healthy volunteers, the FFV and PV revealed good to excellent agreement between both software packages, with only minimal systematic bias and low absolute and relative differences. Correlation analyses revealed very good correlations globally between both software solutions. For the FFV (Supplementary Figure 1), there were no significant correlations between both software packages in plane 1. The other planes revealed very good correlations. For PV (Supplementary Figure 2), there were also no significant correlations between both software packages in plane 1. The other planes revealed very good correlations.

**Table 2:** Plane-wise comparison of the forward flow volumes between both software packages.

Plane	Forward flow volume in software 1 (ml)		Forward flow volume in software 2 (ml)		Absolute and relative differences in forward flow volume between both software packages		ICC	
	Healthy volunteers	Patients	Healthy volunteers	Patients	Healthy volunteers	Patients	Healthy volunteers	Patients
Plane 1	67.0 ± 10.8	—	66.9 ± 8.7	—	0.1 ± 5.4 ml	—	0.93	—
					−0.3 ± 8%			
Plane 2	60.7 ± 10.2	56.6 ± 12.1	62.9 ± 9.5	64.9 ± 13.3	−2.2 ± 2.8 ml	−8.3 ± 9.9 ml	0.97	0.75
					−3.8 ± 4.6%	−13.8 ± 17.4%		
Plane 3	57.9 ± 11.3	65.2 ± 10.3	60.1 ± 11.3	67.0 ± 12.8	−2.2 ± 2.9 ml	−1.8 ± 6.5 ml	0.98	0.91
					−4.8 ± 4.6%	−2.2 ± 9.2%		
Plane 4	42.2 ± 6.3	51.2 ± 14.0	43.4 ± 5.5	46.4 ± 13.4	−1.2 ± 2.9 ml	4.7 ± 9.1 ml	0.93	0.85
					−3.0 ± 6.9%	11.5 ± 30.1%		
Plane 5	40.8 ± 6.8	49.9 ± 12.9	42.3 ± 5.8	46.1 ± 13.6	−1.6 ± 2.0 ml	3.8 ± 9.6 ml	0.96	0.84
					−4.2 ± 4.9%	10.1 ± 30.6%		
Plane 6	39.9 ± 7.0	43.0 ± 10.9	42.1 ± 6.7	39.2 ± 11.6	−2.3 ± 2.4 ml	3.8 ± 7.6 ml	0.95	0.85
					−5.7 ± 6.0%	12.0 ± 31.9%		

ICC, intraclass correlation coefficient.

Values are presented as mean ± standard deviation.

**Table 3:** Plane-wise comparison of peak velocities between both software packages.

Plane	Peak velocity in software 1 (m/s)		Peak velocity in software 2 (m/s)		Absolute and relative differences in peak velocity between both software packages		ICC	
	Healthy volunteers	Patients	Healthy volunteers	Patients	Healthy volunteers	Patients	Healthy volunteers	Patients
Plane 1	1.2 ± 0.1	—	1.2 ± 0.1	—	0.04 ± 0.1 m/s	—	0.81	—
					3.4 ± 7.7%			
Plane 2	0.9 ± 0.2	1.1 ± 0.1	0.9 ± 0.2	1.3 ± 0.2	−0.03 ± 0.1 m/s	−0.2 ± 0.2 m/s	0.96	0.33
					−3.2 ± 6.8%	−19.7 ± 12.7%		
Plane 3	0.9 ± 0.2	1.0 ± 0.2	0.9 ± 0.2	1.2 ± 0.2	−0.1 ± 0.1 m/s	−0.2 ± 0.2 m/s	0.96	0.72
					−9.3 ± 6.1%	−18.7 ± 15.3%		
Plane 4	0.9 ± 0.2	0.9 ± 0.3	0.9 ± 0.2	1.0 ± 0.3	−0.03 ± 0.04 m/s	−0.1 ± 0.1 m/s	0.99	0.95
					−3.6 ± 4.1%	−7.8 ± 9.9%		
Plane 5	1.0 ± 0.2	0.8 ± 0.2	1.0 ± 0.2	0.8 ± 0.2	−0.02 ± 0.02 m/s	−0.03 ± 0.07 m/s	0.99	0.98
					−1.6 ± 2.1%	−3.5 ± 7.9%		
Plane 6	1.1 ± 0.2	0.7 ± 0.2	1.2 ± 0.2	0.7 ± 0.2	−0.1 ± 0.1 m/s	−0.04 ± 0.08 m/s	0.89	0.95
					−4.6 ± 9.9%	−4.4 ± 9.3%		

ICC, intraclass correlation coefficient.

Values are presented as mean ± standard deviation.

In patients, there was also good to excellent agreement for the FFV, with the least comparability in the ascending aorta. The latter also applies for PV, in which excellent agreement could be demonstrated in the aortic arch and descending aorta and only poor to moderate agreement in the ascending aorta. Correlation analyses showed very good correlations between software 1 and 2 globally, as in healthy volunteers. For the FFV (Supplementary Figure 1), there were no significant correlations in plane 2. The other planes revealed good to very good correlations. For PV (Supplementary Figure 2), there were also no significant correlations between both software packages in plane 2. The other planes revealed good to very good correlations.

### 3.3 Maximum wall shear stress

**Figure 5:**
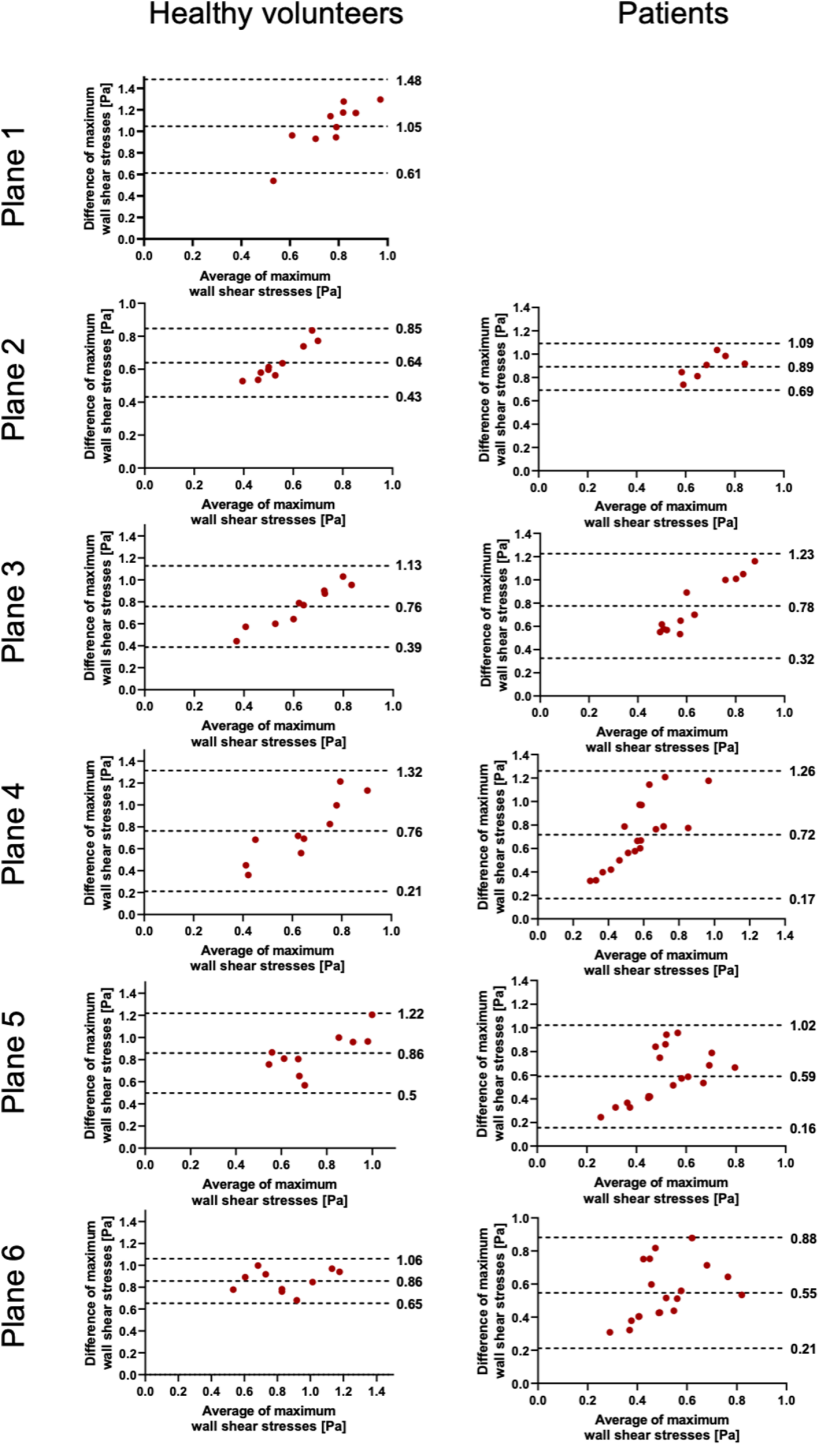
Bland–Altman plots for the plane-wise comparison of maximum wall shear stresses for healthy volunteers and patients. The upper and lower dotted bold lines indicate the 95% limits of agreement. The middle dotted bold line indicates the mean difference.

**Figure 6:**
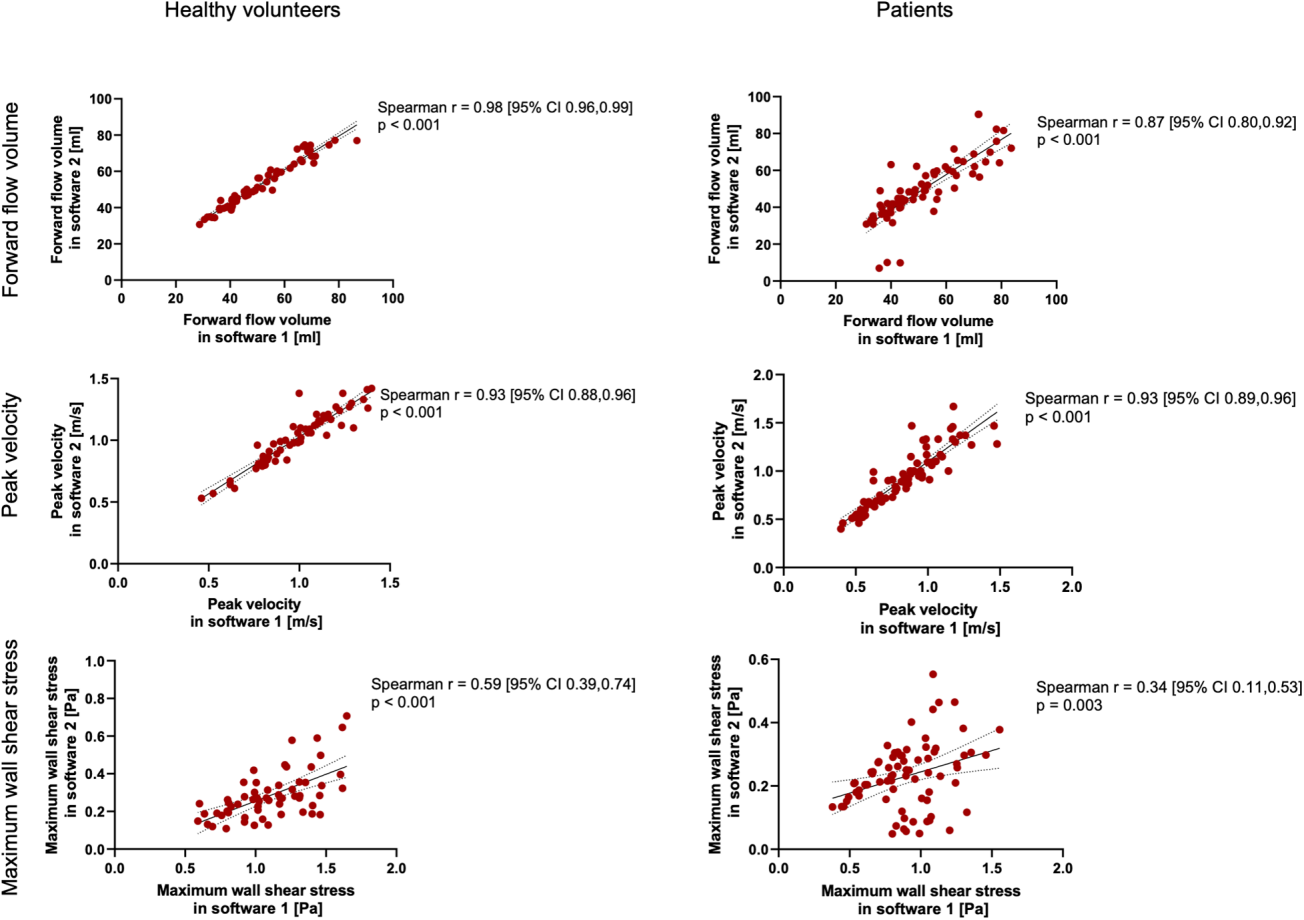
Spearman's correlation analysis for healthy volunteers and patients across all planes combined. The solid line indicates the best-fit line of a simple linear regression model. The dotted lines indicate the 95% confidence bands of the best-fit lines.

The results of WSS are displayed in Table 4 and Figures 5, 6. In contrast to the FFV and PV, WSS analysis showed no to poor agreement, with a systematic bias between both software packages. There was only a poor to moderate correlation globally between both software packages. Correlation analyses for the individual planes are displayed in Supplementary Figure 3. There were no significant correlations in planes 1, 4, and 5 in healthy volunteers and in all planes in patients. There were very good correlations in planes 2, 3, and 6 in healthy volunteers.

**Table 4:** Plane-wise comparison of maximum wall shear stress between both software packages.

Plane	Maximum wall shear stress in software 1 (Pa)		Maximum wall shear stress in software 2 (Pa)		Absolute and relative differences in maximum wall shear stress between both software packages		ICC	
	Healthy volunteers	Patients	Healthy volunteers	Patients	Healthy volunteers	Patients	Healthy volunteers	Patients
Plane 1	1.3 ± 0.2	—	0.2 ± 0.1	—	1.0 ± 0.2 Pa	—	0.01	—
					135.8 ± 16.9%			
Plane 2	0.9 ± 0.2	1.1 ± 0.1	0.2 ± 0.1	0.2 ± 0.1	0.6 ± 0.1 Pa	0.9 ± 0.1 Pa	0.07	0.03
					118.6 ± 7.7%	129.7 ± 12.0%		
Plane 3	1.0 ± 0.2	1.0 ± 0.3	0.2 ± 0.1	0.3 ± 0.05	0.8 ± 0.2 Pa	0.8 ± 0.2 Pa	0.09	0.04
					121.8 ± 9.3%	120.0 ± 14.4%		
Plane 4	1.0 ± 0.3	0.9 ± 0.3	0.3 ± 0.1	0.2 ± 0.1	0.8 ± 0.3 Pa	0.7 ± 0.3 Pa	0.05	0.06
					117.3 ± 22.9%	124.0 ± 28.3%		
Plane 5	1.2 ± 0.2	0.8 ± 0.2	0.3 ± 0.1	0.2 ± 0.1	0.9 ± 0.2 Pa	0.6 ± 0.2 Pa	0.10	0.07
					116.3 ± 22.1%	114.2 ± 34.6%		
Plane 6	1.3 ± 0.2	0.8 ± 0.2	0.4 ± 0.2	0.2 ± 0.1	0.9 ± 0.1 Pa	0.5 ± 0.2 Pa	0.19	0.12
					107.5 ± 30.3%	109.5 ± 32.7%		

ICC, intraclass correlation coefficient.

Values are presented as mean ± standard deviation.

## 4 Discussion

The main findings of our study comparing the results of basic and advanced hemodynamic parameters of 4D flow CMR examinations using two commercially available software packages in healthy volunteers and patients are the following: there was good to excellent agreement for the FFV and PV in healthy study participants and patients except for the ascending aorta in the PV. There was a very good correlation globally between both software packages. However, when comparing the maximum WSS per plane, there was no to poor agreement along the entire thoracic aorta, with systematically higher results in software 1 than in software 2. To our knowledge, this is the first study examining the software comparability of 4D flow CMR dataset analyzation in patients with cardiovascular diseases for PV and WSS.

Oechtering et al. also compared the two software packages that we used in our study in addition to two others (6). In their study on eight healthy volunteers, FFV analysis yielded only moderate agreement and peak velocity analysis only poor agreement, which contrasts with our results with excellent and good to excellent agreement for the FFV and PV in healthy participants, respectively. These differences might be due to several reasons. First, we used different software versions. A second explanation might be that we could manually correct the vessel wall segmentation in software 2, in contrast to Oechtering et al., for whom this was not possible.

Compared with healthy volunteers, we found only moderate agreement in the PV in the ascending aorta. Owing to persistent aliasing despite correction in the majority of cases, we could only analyze a fraction of patients at the mid-ascending aorta. The observed differences might be explained by the known variation of this parameter (6, 13). Another reason might be the existence of non-laminar flow patterns in AS (8). This is the reason why some authors argue against the plane-wise analysis of parameters susceptible to noise, as it is the case in peak velocity (6). van Ooij et al. introduced a pixel-wise analysis rather than plane-wise analysis and compared the reproducibility of WSS in volunteers, and the results showed small absolute differences and low coefficients of variation between test–retest examinations several days apart (14). This option, however, is not available in both software packages used. In addition, it is known that the ascending aorta is the area with the largest volume change and motion over the cardiac cycle due to its compliance and is therefore the hardest to contour (13).

There was no to poor agreement regarding maximum WSS per plane, which was also demonstrated by Oechtering et al. (6). Software 1 revealed systematically higher values in the range of three to fourfold differences compared with software 2. In software 1, the results of 90 segments per phase per plane for WSS are provided. We calculated an average of 90 segments for every cardiac phase and defined the maximum WSS as the highest value out of all phases accordingly. In software 2, the maximum WSS is the highest WSS value over the entire analysis plane and out of all phases, but still yielded lower WSS values than software 1. However, despite several inquiries about the basis of the calculation of WSS, both vendors did not reveal their respective calculation approach. Except for literature references for one software package provided in the user manual, no specific and detailed information in this regard is provided in the manuals (13, 15–17). Ultimately, an unclear definition of the WSS value makes comparisons between values obtained using different software packages invalid. In addition to potential differences in the calculations, another possible major influencing factor is certainly the definition of vessel wall boundaries (13, 18, 19). As described, WSS is much more dependent on segmentation contours than peak velocity or mean flow, as the basis of WSS calculation is based on the local velocity derivative at the boundary (18). Differences in WSS between different software solutions might preclude intra-individual comparison during follow-up, especially if obtained at different scanning sites that use different software packages. In addition, a comparison of the study results of participating centers in possible multicenter studies might be difficult if different software packages are used.

In summary, in contrast to flow parameters, which agreed well between both software packages examined in our study, WSS values were not comparable between both software solutions. Several reasons might explain the systematic difference observed in our study.

### 4.1 Limitations

Several limitations apply to our study. Our study cohort and sample size were relatively small, which might impact the statistical power and generalizability of our findings. The sample consisted only of a retrospective dataset that was obtained in a monocentric setting. We only compared two software solutions and focused on three quantitative parameters. Future studies should therefore concentrate on a prospective multicenter design with adequately powered study samples investigating multiple software packages, both those that are commercially available and those that are research solutions. We had to exclude several planes due to aliasing.

## 5 Conclusion

A comparison of different 4D flow CMR software packages in healthy volunteers and 20 patients with aortic valve pathologies revealed good to very good agreement for flow parameters, e.g., FFV and PV, except for the ascending aorta in the latter. There was no agreement for WSS values, with a systematic difference. For potentially larger prospective studies, standardization not only in the scanning parameters but also regarding post-processing is necessary.

## Data Availability

The data analyzed in this study are subject to the following licenses/restrictions: the datasets for this study are not publically available due to German data protection laws. Requests to access these datasets should be directed to jeanette.schulz-menger@charite.de.

## Supplementary material

Supplementary Figure 1 Spearman's correlation analysis of forward flow volumes for healthy volunteers and patients across each individual plane. The solid line indicates the best-fit line of a simple linear regression model. The dotted lines indicate the 95% confidence bands of the best-fit lines.

Supplementary Figure 2 Spearman's correlation analysis of peak velocities for healthy volunteers and patients across each individual plane. The solid line indicates the best-fit line of a simple linear regression model. The dotted lines indicate the 95% confidence bands of the best-fit lines.

Supplementary Figure 3 Spearman's correlation analysis of maximum wall shear stress for healthy volunteers and patients across each individual plane. The solid line indicates the best-fit line of a simple linear regression model. The dotted lines indicate the 95% confidence bands of the best-fit lines.

## References

[1] Paddock S Tsampasian V Assadi H Mota BC Swift AJ Chowdhary A Clinical translation of three-dimensional scar, diffusion tensor imaging, four-dimensional flow, and quantitative perfusion in cardiac MRI: a comprehensive review. Front Cardiovasc Med. (2021) 8:682027. 10.3389/fcvm.2021.682027 34307496 PMC8292630

[2] Chowdhary A Garg P Das A Nazir MS Plein S. Cardiovascular magnetic resonance imaging: emerging techniques and applications. Heart. (2021) 107:697–704. 10.1136/heartjnl-2019-315669 33402364 PMC7611390

[3] Wiesemann S Schmitter S Demir A Prothmann M Schwenke C Chawla A Impact of sequence type and field strength (1.5, 3, and 7T) on 4D flow MRI hemodynamic aortic parameters in healthy volunteers. Magn Reson Imaging. (2021) 85:721–33. 10.1002/mrm.28450 32754969

[4] Demir A Wiesemann S Erley J Schmitter S Trauzeddel RF Pieske B Traveling volunteers: a multi-vendor, multi-center study on reproducibility and comparability of 4D flow derived aortic hemodynamics in cardiovascular magnetic resonance. J Magn Reson Imaging. (2022) 55:211–22. 10.1002/jmri.27804 34173297

[5] Zange L Muehlberg F Blaszczyk E Schwenke S Traber J Funk S Quantification in cardiovascular magnetic resonance: agreement of software from three different vendors on assessment of left ventricular function, 2D flow and parametric mapping. J Cardiovasc Magn Reson. (2019) 21:12. 10.1186/s12968-019-0522-y 30786898 PMC6383230

[6] Oechtering TH Nowak A Sieren MM Stroth AM Kirschke N Wegner F Repeatability and reproducibility of various 4D flow MRI postprocessing software programs in a multi-software and multi-vendor cross-over comparison study. J Cardiovasc Magn Reson. (2023) 25:22. 10.1186/s12968-023-00921-4 36978131 PMC10052852

[7] Burkhardt BEU Kellenberger CJ Callaghan FM Buechel ERV Geiger J. Flow evaluation software for four-dimensional flow MRI: a reliability and validation study. Radiol Med. (2023) 128:1225–35. 10.1007/s11547-023-01697-4 37620674 PMC10547653

[8] Wiesemann S Trauzeddel RF Musa A Hickstein R Mayr T von Knobelsdorff-Brenkenhoff F Changes of aortic hemodynamics after aortic valve replacement—a four dimensional flow cardiovascular magnetic resonance follow up study. Front Cardiovasc Med. (2023) 10:1071643. 10.3389/fcvm.2023.1071643 36865891 PMC9971963

[9] Bernstein MA Zhou XJ Polzin JA King KF Ganin A Pelc NJ Concomitant gradient terms in phase contrast MR: analysis and correction. Magn Reson Med. (1998) 39:300–8. 10.1002/mrm.1910390218 9469714

[10] Dyverfeldt P Bissell M Barker AJ Bolger AF Carlhall CJ Ebbers T 4D Flow cardiovascular magnetic resonance consensus statement. J Cardiovasc Magn Reson. (2015) 17:72. 10.1186/s12968-015-0174-5 26257141 PMC4530492

[11] Walker PG Cranney GB Scheidegger MB Waseleski G Pohost GM Yoganathan AP. Semiautomated method for noise reduction and background phase error correction in MR phase velocity data. J Magn Reson Imaging. (1993) 3:521–30. 10.1002/jmri.1880030315 8324312

[12] Koo TK Li MY. A guideline of selecting and reporting intraclass correlation coefficients for reliability research. J Chiropr Med. (2016) 15:155–63. 10.1016/j.jcm.2016.02.012 27330520 PMC4913118

[13] Stalder AF Russe MF Frydrychowicz A Bock J Hennig J Markl M. Quantitative 2D and 3D phase contrast MRI: optimized analysis of blood flow and vessel wall parameters. Magn Reson Med. (2008) 60:1218–31. 10.1002/mrm.21778 18956416

[14] van Ooij P Powell AL Potters WV Carr JC Markl M Barker AJ. Reproducibility and interobserver variability of systolic blood flow velocity and 3D wall shear stress derived from 4D flow MRI in the healthy aorta. J Magn Reson Imaging. (2016) 43:236–48. 10.1002/jmri.24959 26140480 PMC4807608

[15] Potters WV van Ooij P Marquering H vanBavel E Nederveen AJ. Volumetric arterial wall shear stress calculation based on cine phase contrast MRI. J Magn Reson Imaging. (2015) 41:505–16. 10.1002/jmri.24560 24436246

[16] Sui B Gao P Lin Y Qin H Liu L Liu G. Noninvasive determination of spatial distribution and temporal gradient of wall shear stress at common carotid artery. J Biomech. (2008) 41:3024–30. 10.1016/j.jbiomech.2008.07.026 18805528

[17] Petersson S Dyverfeldt P Ebbers T. Assessment of the accuracy of MRI wall shear stress estimation using numerical simulations. J Magn Reson Imaging. (2012) 36:128–38. 10.1002/jmri.23610 22336966

[18] Markl M Wallis W Harloff A. Reproducibility of flow and wall shear stress analysis using flow-sensitive four-dimensional MRI. J Magn Reson Imaging. (2011) 33:988–94. 10.1002/jmri.22519 21448968

[19] Frydrychowicz A Stalder AF Russe MF Bock J Bauer S Harloff A Three-dimensional analysis of segmental wall shear stress in the aorta by flow-sensitive four-dimensional-MRI. J Magn Reson Imaging. (2009) 30:77–84. 10.1002/jmri.21790 19557849

