# Supplementary figures and images for: The influence of post-processing software on quantitative results in 4D flow cardiovascular magnetic resonance examinations
Source: Front Cardiovasc Med. 2024 Sep 27;11:1465554. doi: 10.3389/fcvm.2024.1465554 (PMC11467864; doi:10.3389/fcvm.2024.1465554)

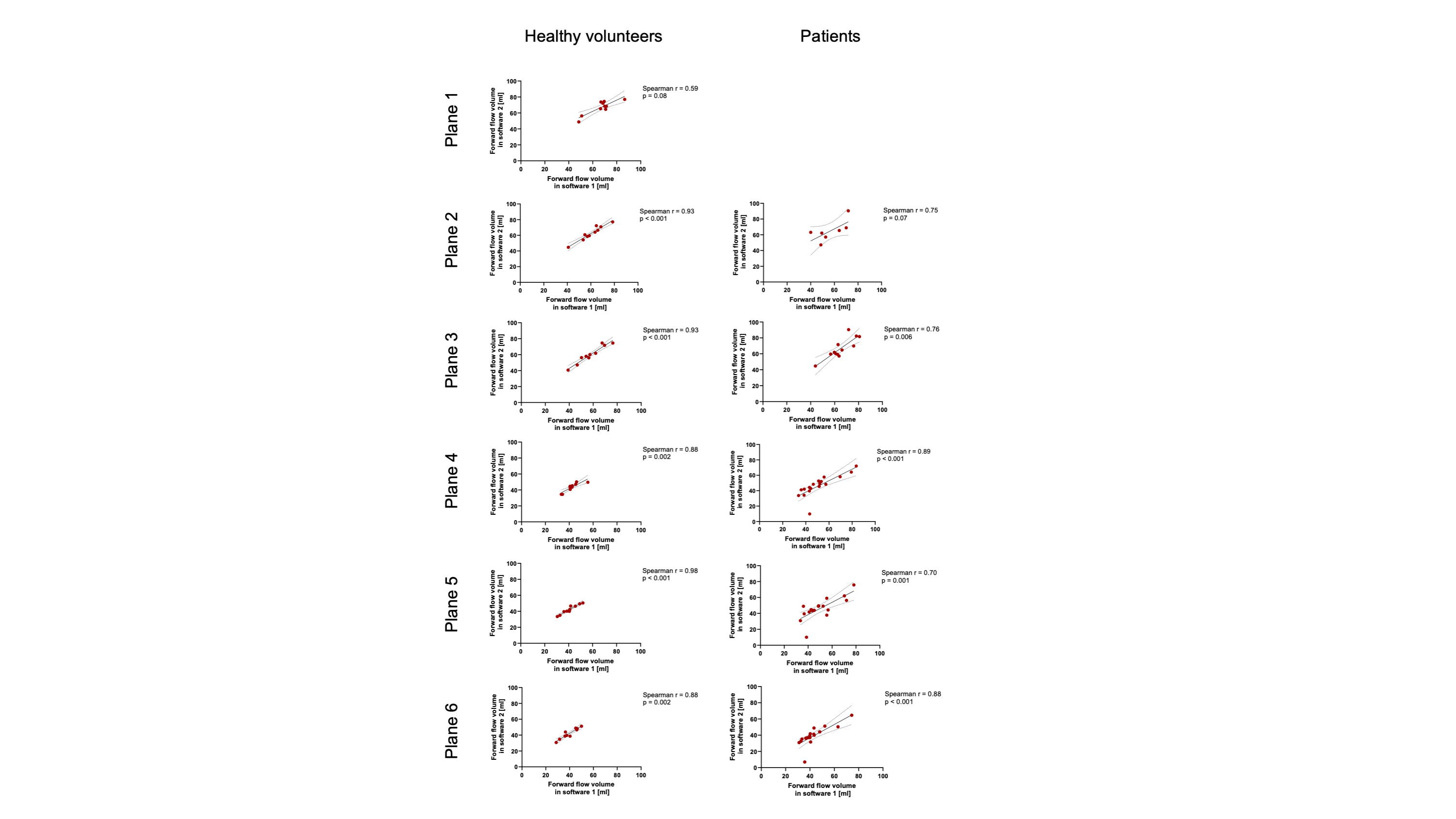

Supplement: Supplementary Figure 1 — Spearman's correlation analysis of forward flow volumes for healthy volunteers and patients across each individual plane. The solid line indicates the best-fit line of a simple linear regression model. The dotted lines indicate the 95% confidence bands of the best-fit lines. [file Image1.tiff]

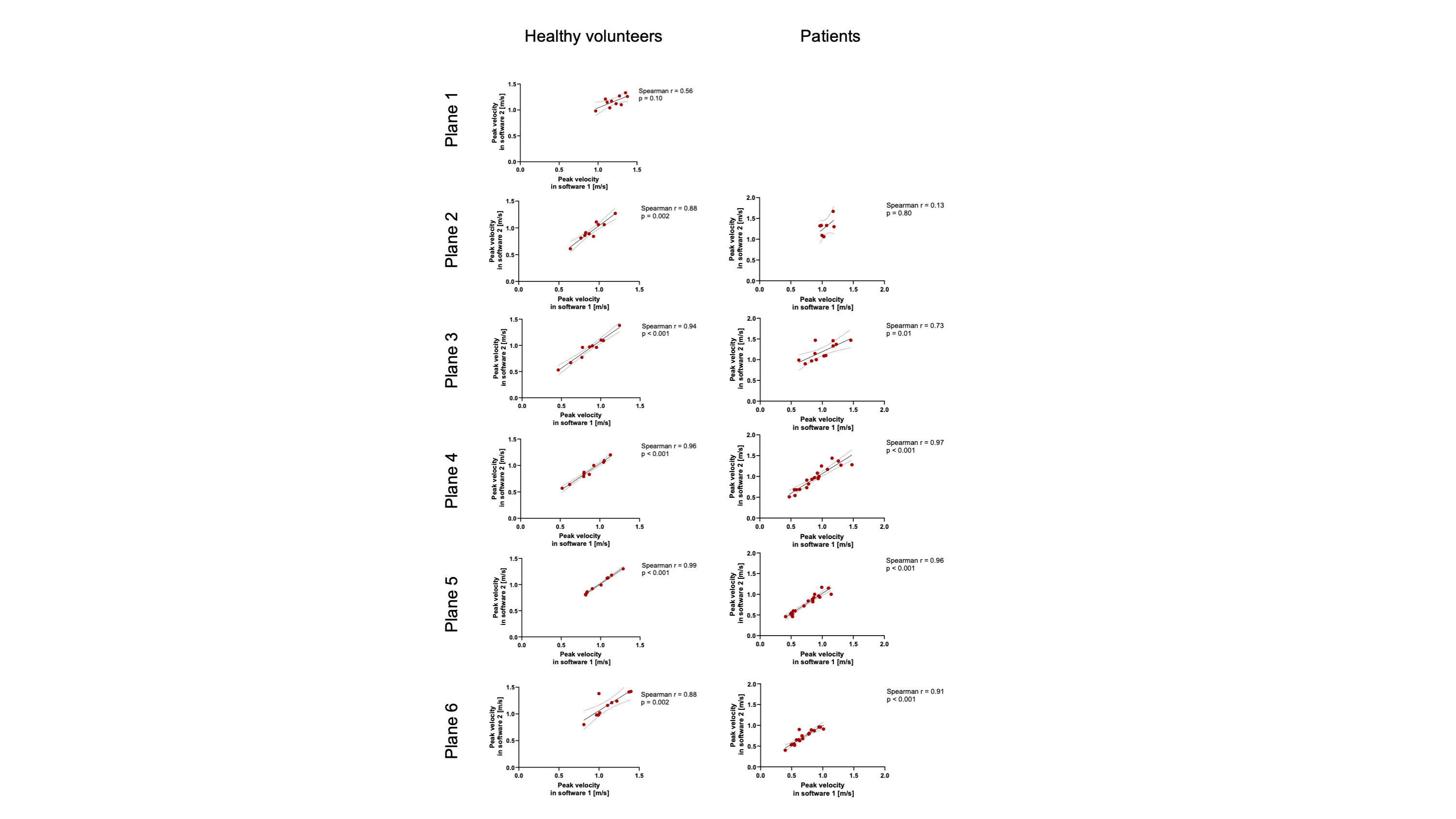

Supplement: Supplementary Figure 2 — Spearman's correlation analysis of peak velocities for healthy volunteers and patients across each individual plane. The solid line indicates the best-fit line of a simple linear regression model. The dotted lines indicate the 95% confidence bands of the best-fit lines. [file Image2.tiff]

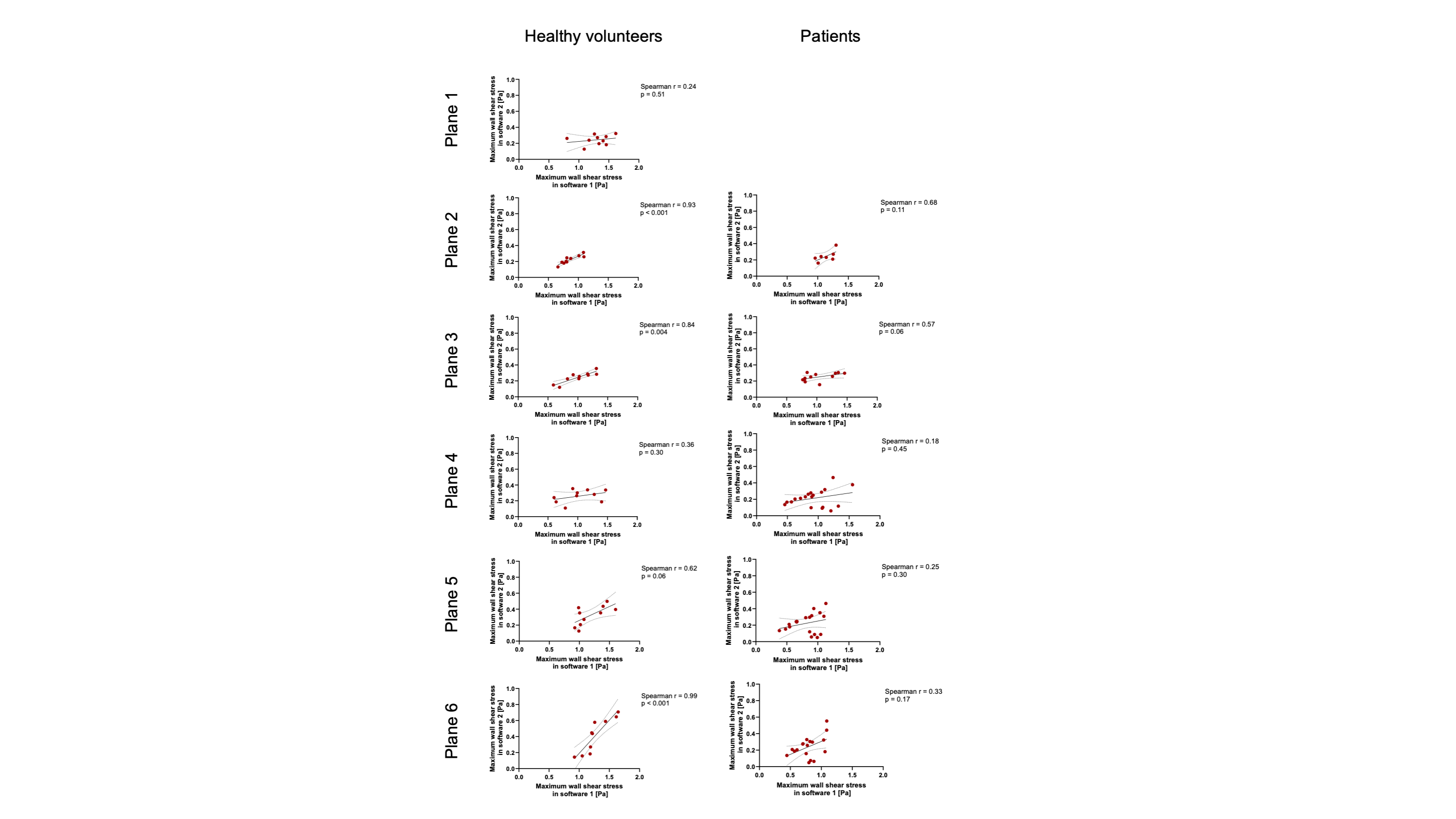

Supplement: Supplementary Figure 3 — Spearman's correlation analysis of maximum wall shear stress for healthy volunteers and patients across each individual plane. The solid line indicates the best-fit line of a simple linear regression model. The dotted lines indicate the 95% confidence bands of the best-fit lines. [file Image3.tiff]
